# Metal-Induced Amide Deprotonation and Binding Typical for Cu(II), Not Possible for Zn(II) and Fe(II)

**DOI:** 10.1021/acs.inorgchem.5c00672

**Published:** 2025-03-26

**Authors:** Silvia Leveraro, Valentyn Dzyhovskyi, Kinga Garstka, Agnieszka Szebesczyk, Fabio Zobi, Denise Bellotti, Kamila Stokowa-Sołtys, Maurizio Remelli, Magdalena Rowińska-Żyrek

**Affiliations:** † Faculty of Chemistry, University of Wroclaw, ul. F. Joliot-Curie 14, 50-383 Wroclaw, Poland; ‡ Department of Chemical, Pharmaceutical and Agricultural Sciences, University of Ferrara, Via Luigi Borsari 46, 44121 Ferrara, Italy; § Institute of Health Sciences, University of Opole, Katowicka str. 68, 45-060 Opole, Poland; ∥ Department of Chemistry, Fribourg University, Chemin Du Musée 9, 1700 Fribourg, Switzerland

## Abstract

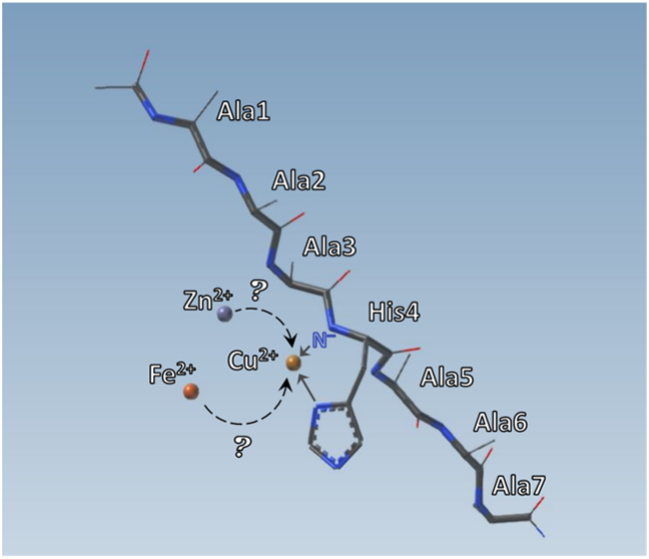

Amide groups of the peptide backbone are very weak acids. In fact, their deprotonation in water solution is not a phenomenon usually observed in the measuring range of a glass electrode unless the proton is displaced by a metal such as Cu(II) or Ni(II). Other metals are not usually expected to deprotonate and bind to amide nitrogens, although, lately, some controversies have started to arise in the literature, suggesting that Zn(II) and Fe(II) may be capable of doing so. In order to clarify this phenomenon, we chose to study simple metal–peptide systems with Ala-to-Pro mutations, which excluded further amides from binding. A comparison of the metal-binding modes of Ac-AAAHAAA-NH_2_, Ac-AAPHAAA-NH_2_, and Ac-AAPHPAA-NH_2_ complexes with Cu(II), Zn(II), and Fe(II) is a simple and elegant way of showing that neither Zn(II) nor Fe(II) is able to deprotonate and bind to amide nitrogens.

## Abstract 2

Amide groups of peptide backbone are very weak acids.

## Introduction

Metal–protein interactions are an intensively studied field and can be regarded as one of the main pillars of bioinorganic chemistry. In particular, the knowledge of the peculiarities of Zn(II) and Cu(II) binding to proteins and peptides is a topic that has been expanding for several decades,^1−7^ while the studies on the thermodynamics of Fe(II) to peptide binding have only recently gained some attention, and the data available on Fe(II)–peptide interactions is very sparse.^8−11^

The amide dissociation constant (p*K*_a_) depends on its chemical environment, usually reaching values in the range of 15–18.^12^ Cu(II) and Ni(II) ions are known to lower the amide p*K*_a_: in fact, these metal ions can displace the amide proton and bind the amide nitrogen at a relatively low pH value (usually pH > 6.0 for Cu(II) and >9.5 for Ni(II) complexes).^13−15^ Typically, the metal ion anchors on a histidine imidazole ring in the acidic pH range; at a higher pH, it deprotonates and binds the nitrogen atom of the amide group that belongs to the same histidine, i.e., the amide group preceding the His residue in the N-terminal direction. At higher pH, further preceding amide groups can be involved in complexation, forming thermodynamically stable five-membered and six-membered fused chelate rings.^16−18^

Proline is the only natural amino acid having a secondary amino nitrogen: proline is technically an imino acid. Its presence in the peptide chain results in a peptide bond that does not contain a replaceable amide proton. As a consequence, the introduction of proline in the peptide sequence serves as a so-called “breaking point”, making the typical stepwise coordination of consecutive amide nitrogens impossible.^19^ It is worth noting that, in the presence of a “breaking proline” before histidine, i.e., in the N-terminal direction, the deprotonation/coordination of amide nitrogens by the metal ion can proceed in the opposite (C-terminal) direction, as it happens for the Prion protein,^20^ although the formed metal chelates have reduced stability.

In recent years, contradictory opinions have started to emerge about the capability of other divalent metal ions to bind amide nitrogens. Intrigued by several literature discussions on the possibility of Zn(II)-induced amide deprotonation and binding^21−23^ and by our own preliminary findings on the possible Fe(II)–amide binding,^8,9^ we wanted to understand if Zn(II) and/or Fe(II) are indeed able to deprotonate amide nitrogens. We decided to study metal complexes of a simple model system based on an N-terminally and C-terminally protected ligand with one histidine only (AHA, (Ac-AAAHAAA-NH_2_)). We substituted the neighboring alanines with prolines (PHA (Ac-AAPHAAA-NH_2_) and PHP (Ac-AAPHPAA-NH_2_)) in order to make sure that amide coordination will not proceed in the N-terminal or C-terminal direction.

## Results and Discussion

Under the experimental conditions employed here, only variously protonated mononuclear complexes with a metal/ligand stoichiometry of 1:1 have been detected by potentiometry and mass spectrometry. Precipitation of copper hydroxide has been observed only in the case of copper with the PHP peptide around pH 7.5, while precipitation of iron hydroxide has been observed with all three peptides around pH 9.5.

### Ligand Protonation

For each peptide, the obtained protonation *macro-*constants are reported in Table 1; the complete set of thermodynamic parameters, obtained by potentiometry and calorimetry, is shown in Table S1. The L symbol indicates the ligand in its unprotonated form, i.e., when all its dissociable protons (in the explored pH range) have been released. The investigated peptides **AHA** (Ac-AAAHAAA-NH_2_), **PHA** (Ac-AAPHAAA-NH_2_), and **PHP** (Ac-AAPHPAA-NH_2_) have the N- and C-terminal ends modified through acetylation and amidation, respectively. Each of them presents only one deprotonable site, represented by the histidine residue. Once completely deprotonated, the ligands are neutral (L). All of the thermodynamic parameters concerning ligand protonation equilibria are in good agreement with literature values for similar systems^24^ and show reasonable similarities among the considered peptides.

**Table 1:** Protonation Constant (*K*) for the Investigated Ligands (**AHA**, **PHA**, and **PHP**) at *T* = 298 K and *I* = 0.1 M (NaCl)^a^

	species	log *K*	residue
Ac-AAAHAAA-NH_2_ (**AHA**)	**HL**^**+**^	6.47(1)	His
Ac-AAPHAAA-NH_2_ (**PHA**)	**HL**^**+**^	6.43(1)	His
Ac-AAPHPAA-NH_2_ (**PHP**)	**HL**^**+**^	6.56(2)	His

a Standard deviations on the last significant digit are listed in parentheses.

### Cu(II) Complexes

The investigated systems are able to form Cu(II) complexes in a stoichiometric ratio of 1:1 under the employed experimental conditions (Tables 2 and S1 in SI). The identified MS signals align closely with equimolar Cu(II) complexes (*m*/*z* = 343.12, 356.13, and 369.14, *z* = 2+ for the AHA, PHA, and PHP ligands, respectively); the experimental signals were compared with the corresponding simulated isotopic patterns, showing perfect alignment (Figures S1–S3 in SI). Furthermore, all measured mass spectra displayed signals corresponding to the ligands and their sodium or potassium adducts.

**Table 2:** Equilibrium Constants and the Proposed Coordination Modes for Cu(II) Complexes at *T* = 298 K and *I* = 0.1 (NaCl)^a^

	species	log β	p*K*_a_	equatorial coordination
Ac-AAAHAAA-NH_2_ (**AHA**)	**[CuL]**^**2+**^	3.44(6)	6.07	N_Im_
	**[CuH**_–**1**_**L]**^**+**^	–2.63(3)	6.14	N_Im_, N^–^
	**[CuH**_–**2**_**L]**	–8.77(1)	8.68	N_Im_, 2N^–^
	**[CuH**_–**3**_**L]**^–^	–17.45(2)		N_Im_, 3N^–^
Ac-AAPHAAA-NH_2_ (**PHA**)	**[CuL]**^**2+**^	3.56(4)		N_Im_
	**[CuH**_–**2**_**L]**	–9.65(2)	9.73	2N^–^
	**[CuH**_–**3**_**L]**^–^	–19.38(2)		3N^–^
Ac-AAPHPAA-NH_2_ (**PHP**)	**[CuL]**^**2+**^	3.81(5)		N_Im_
	**[CuH**_–**2**_**L]**	–9.66(3)		N_Im_, N^–^, OH^–^

a Values in parentheses are standard deviations on the last significant digit.

**Figure 1:**
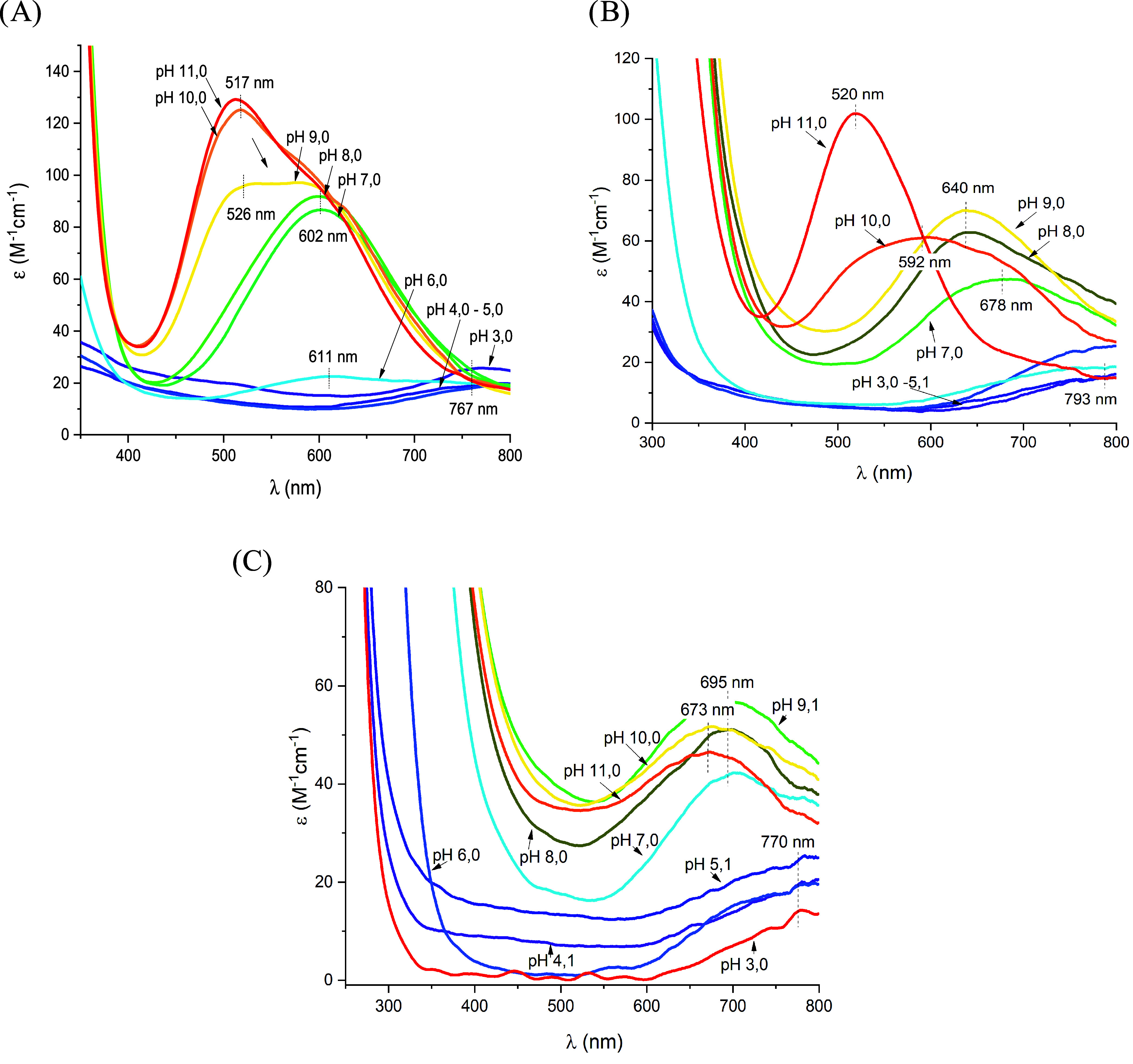
UV–vis absorption spectra of Cu(II) complexes with (A) **AHA**, (B) **PHA**, and (C) **PHP** at different pH values: M:L 0.9:1, *C*_M_ = 0.45 × 10^–3^ M, optical path 1 cm. The wavelength of the maximum absorption is reported for each vis-absorption spectrum.

**Figure 2:**
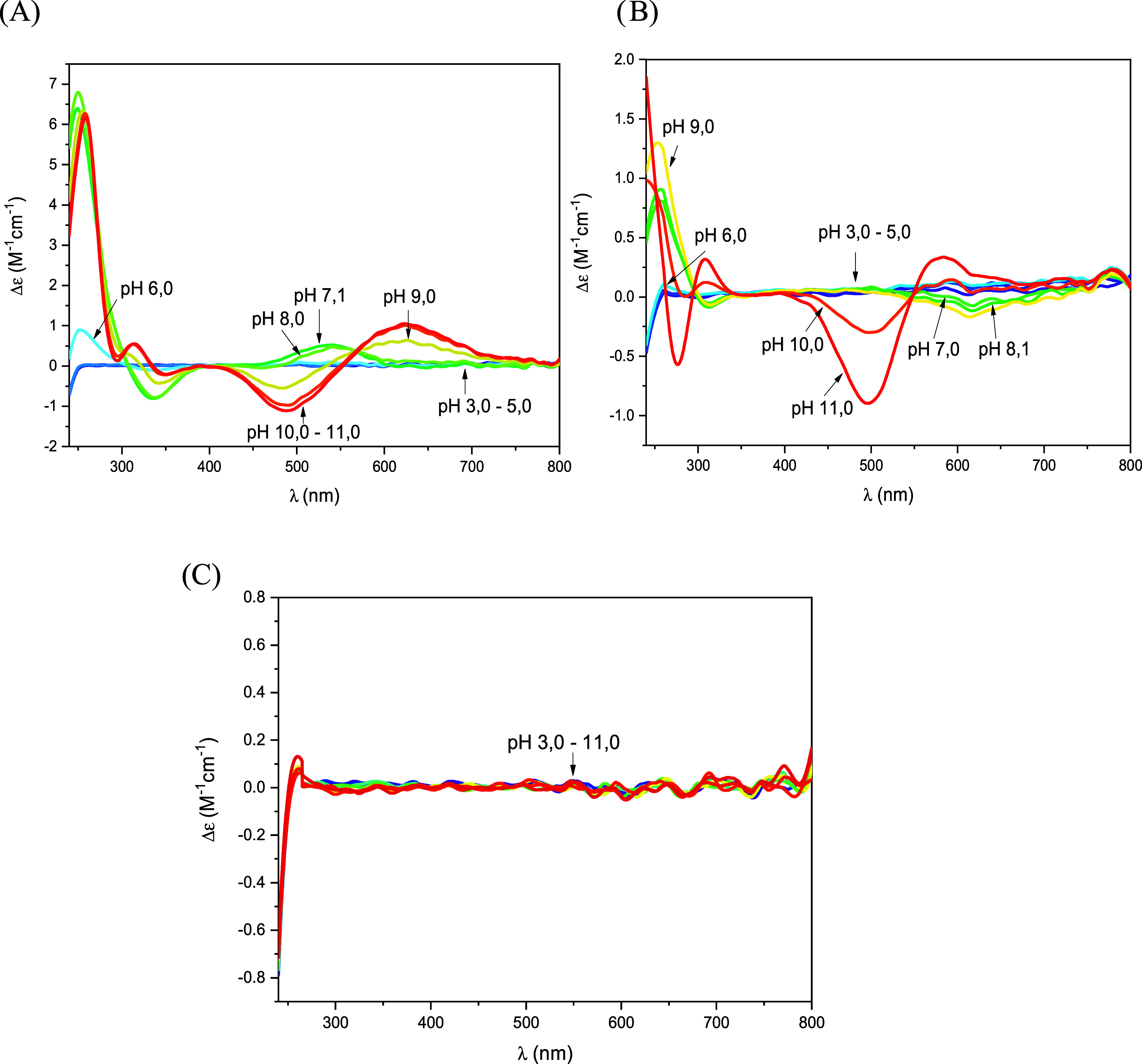
CD spectra of Cu(II) complexes with (A) **AHA**, (B) **PHA**, and (C) **PHP** at different pH values: M:L ratio 0.9:1, *C*_M_ = 0.44 × 10^–3^ M, optical path 1 cm.

**Figure 3:**
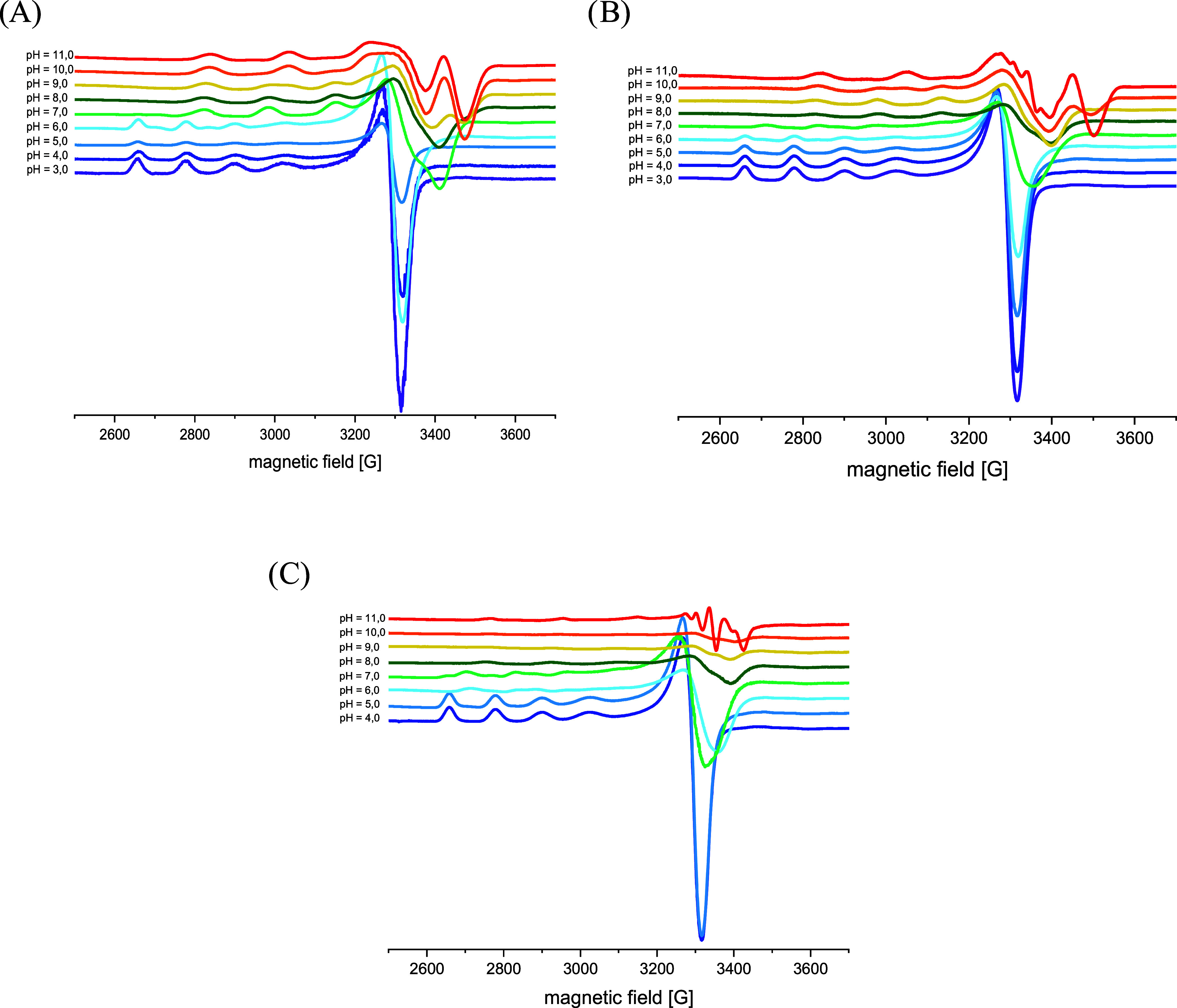
X-band EPR spectra of frozen solution (77 K) of Cu(II) complexes with (A) **AHA**, (B) **PHA**, and (C) **PHP** at different pH values: *I* = 0.1 M (NaCl), M:L ratio 0.9:1, *C*_M_ = 2 × 10^–3^ M.

The formation of copper complexes in solution begins around pH 4 for all of the studied ligands (see distribution diagrams reported in Figures S4–S6 in SI). The first complex detected by potentiometry is always [CuL]^2+^: the stoichiometry of this species indicates that histidine is already deprotonated and thus coordinated to copper. This species is 1N (N_Im_), with the other coordination positions of the metal ion being saturated by water molecules. The complex [CuL]^2+^ reaches its maximum of formation around pH 5.8 with **AHA** and pH 6.3 with **PHA** and **PHP**. The experimental wavelengths of maximum absorption (λ_max_ = 767 nm for **AHA** at pH 5.0, λ_max_ = 766 nm for **PHA** at pH 6.0, and λ_max_ = 762 nm for **PHP** at pH 6.0; Figure 1) are in good agreement with the expected value for a coordination (1N_Im_) (expected λ_max_ = 760 nm).^12^ Increasing the pH value, in the case of **AHA**, the first and the second amide deprotonate and coordinate copper almost simultaneously: the complex [CuH_–1_L]^+^ is quickly substituted with the [CuH_–2_L] species, which is, by far, the most abundant complex in a solution with pH of 6.3 up to 8.3. These two steps (p*K*_a_ = 6.08 and p*K*_a_ = 6.13; Table 2) are accompanied by a shift in the visible absorption spectra toward shorter wavelengths, thus indicating an increase in the number of coordinated nitrogen atoms around Cu(II) (Figure 1A). The experimental wavelength of the maximum absorption at pH 8 is 602 nm, only a little bit higher than the predicted value for a (N_Im_, 2N^–^) coordination mode (583 nm).^12,25^ Finally, the last detected complex is [CuH_–3_L]^−^, which is characterized by p*K*_a_ = 8.68. The visible spectra in the alkaline pH range suggest the coordination of a third amide. The formation of that species causes a clear blue-shift of the vis-absorption band to λ_max_ = 517 nm, which almost perfectly agrees with the expected literature value (522 nm)^12^ for the (N_Im_, 3N^–^) coordination mode with a square planar geometry. Moreover, the shape and intensity of the CD signal in the pH range 9.0–11.0 (Figure 2A) show a negative Cotton effect at 490 nm and a positive Cotton effect at 624 nm, along with, in the UV region, a positive band at 314 nm (distinctive of a N_amide_^–^ → Cu(II) charge transfer^26^), consistent with the suggested (N_Im_, 3N^–^) coordination mode. In addition, the 4N binding mode for [CuH_–3_L]^−^ is also confirmed by electron paramagnetic resonance (EPR) results at pH 10 (*g*_II_ = 2.19, *A*_II_ = 197; (Figure 3A and Table S2 in SI).^27^ The coordination path described above and the corresponding donor-atom sets are also supported by complex-formation enthalpies (Table S1 in SI), which, in turn, are in excellent agreement with literature data for analogous peptides.^28^ Lastly, on the basis of the wide available literature on Cu(II)/peptide complexes,^20,29−32^ although in the absence of direct evidence, we can reasonably suggest that the progressive amide coordination proceeds in the N-terminal direction, starting from the His anchoring site.

In the case of **PHA**, the [CuH_–1_L]^+^ species has not been detected by potentiometry, while the formation of the [CuH_–2_L] complex was observed, starting from pH 6 and involving almost all of the Cu(II) ions around pH 8. The stoichiometry of this species implies the deprotonation/coordination of two amide nitrogens, necessarily in the C-terminal direction (starting from the His residue to which the metal ion is anchored), due to the presence of proline before His. Indeed, the value of λ_max_ ≈ 640 nm suggests a (2N^–^) coordination mode in the equatorial plane of the complex (expected λ_max_ value: 633 nm);^12^ the imidazole nitrogen, already bound to the metal ion should be shifted to an axial position of the distorted octahedron, justifying the observed red-shift between the experimental and expected λ_max_ values.^12^ Two water molecules complete the equatorial coordination. Finally, at alkaline pH values, a last deprotonation step has been observed with a p*K*_a_ = 9.73; all of the spectroscopic parameters suggest the coordination of a further amide nitrogen (available in the C-terminal direction). In addition, the Δ*H°* value for the formation of the [CuH_–3_L]^−^ species (76 kJ/mol; Table S1 in SI) is practically identical to the corresponding one of AHA (73 kJ/mol; Table S1 in SI), suggesting the same donor-atom set, most likely with the imidazole in the axial position.

As for the last peptide, **PHP**, potentiometric and calorimetric measurements have been performed only up to pH 7.5 due to copper hydroxide precipitation. In this case, the two alanines adjacent to the histidine (positions 3 and 5) have been replaced with prolines, and only one backbone amide is available next to the coordinated histidine in the N-terminal direction. As in the previous peptide, after the coordination of imidazole nitrogen with the formation of the [CuL]^2+^ complex, the expected deprotonation and coordination of the amide nitrogen were not detectable by potentiometry. The species [CuH_–2_L] was instead found to form, derived from the deprotonation of a water molecule and starting from a pH value of 6, with likely coordination (1N_Im_, N^–^, OH^–^). The experimental value of λ_max_ at pH 8 (≈658 nm) is in good agreement with the expected value for the suggested coordination mode (expected λ_max_ = 660 nm).^12^ The 2N binding mode is also confirmed by EPR results (Figure 3C and Table S2 in SI).^27^ The CD spectra (Figure 2C) remain unchanged throughout the entire explored pH range: no signal of the coordinated nitrogens could be detected, but this is not unusual when only one backbone amide is bound to copper. In conclusion, the presence of two proline residues around the metal-binding site provides great structural rigidity, thus hindering the binding of other backbone amides far from the His anchoring site.

### Zn(II) Complexes

The investigated peptides are able to form Zn(II) complexes in a stoichiometric ratio of 1:1 under the employed experimental conditions. The identified MS signals align closely with equimolar Zn(II) complexes (*m*/*z* = 343.12, 356.13, and 369.14, *z* = 2+ for the **AHA**, **PHA**, and **PHP** ligands, respectively; Figures S1–S3 in SI), and they are almost perfectly superimposable to the corresponding simulated isotopic patterns.

Potentiometric results, reported in Table 3 and plotted in the distribution diagrams of Figures S7–S9, show that the three ligands behave in a very similar way toward the Zn(II) ion. In fact, for all of the studied systems, at the most acidic pH values, the free metal ion is prevalent and Zn(II) complexes begin to form only above pH 4. The first detected species is [ZnL]^2+^, in which the histidine residue is deprotonated and bound to the metal ion. Its complex geometry can be tetrahedral,^18,33,34^ where three water molecules complete the Zn(II) coordination sphere. When the pH value is increased, two complexes are formed, [ZnH_–2_L] and [ZnH_–3_L]^−^, most likely derived from the [ZnL]^2+^ complex through the simple release of protons from the coordinated water molecules. All of the thermodynamic results unequivocally demonstrate that the availability or not of backbone amide nitrogens near the His anchoring site makes no difference in the complexing behavior of Zn(II) ion, and the only binding site always is the imidazole side group of histidine. Of particular relevance is the result concerning the formation enthalpy values of the [ZnH_–3_L]^−^ species, formed by the three peptides, which are almost identical, taking into account the standard deviations of 107 kJ/mol for AHA, 103 kJ/mol for PHA, and 106 kJ/mol for PHP (Table S1 in SI). In fact, this is the species formed in the most alkaline pH range; thus, the complex is potentially more prone to involve amide nitrogens in metal coordination.

**Table 3:** Equilibrium Constants and the Proposed Coordination Modes for Zn(II) Complexes at *T* = 298 K and *I* = 0.1 (NaCl)^a^

	species	log β	p*K*_a_	coordination mode
Ac-AAAHAAA-NH_2_ (**AHA**)	**[ZnL]**^**2+**^	3.70(6)		N_Im_
	**[ZnH**_–**2**_**L]**	–12.27(6)	8.02	N_Im_, 2OH^–^
	**[ZnH**_–**3**_**L]**^–^	–20.29(4)		N_Im_, 3OH^–^
Ac-AAPHAAA-NH_2_ (**PHA**)	**[ZnL]**^**2+**^	3.60(1)		N_Im_
	**[ZnH**_–**2**_**L]**	–12.00(1)	8.01	N_Im_, 2OH^–^
	**[ZnH**_–**3**_**L]**^–^	–20.01(8)		N_Im_, 3OH^–^
Ac-AAPHPAA-NH_2_ (**PHP**)	**[ZnL]**^**2+**^	3.70(6)		N_Im_
	**[ZnH**_–**2**_**L]**	–12.13(6)	8.06	N_Im_, 2OH^–^
	**[ZnH**_–**3**_**L]**^–^	–20.19(4)		N_Im_, 3OH^–^

a Values in parentheses are standard deviations on the last significant digit.

### Fe(II) Complexes

Also, the systems containing the Fe(II) ion have been investigated by potentiometry (Table 4 and Figures S10–S12) with the support of mass spectrometry for a confirmation of the stoichiometry of the formed complexes (Figures S1–S3 in SI.) Once again, only mononuclear 1:1 species have been detected, with a degree of protonation depending on the solution pH. Iron hydroxide precipitation was observed at a pH higher than 9.5.

**Table 4:** Equilibrium Constants and the Proposed Coordination Modes for Fe(II) Complexes at *T* = 298 K and *I* = 0.1 M (NaCl)^a^

	species	log β	p*K*_a_	coordination mode
Ac-AAAHAAA-NH_2_ (**AHA**)	**[FeL]**^**2+**^	3.41(6)	7.75	N_Im_
	**[FeH**_–**1**_**L]**^**+**^	–4.34(3)		N_Im_, OH^–^
	**[FeH**_–**3**_**L]**^–^	–22.28(3)	10.25	N_Im_, 3OH^–^
	**[FeH**_–**4**_**L]**^**2**–^	–32.53(3)		N_Im_, 4OH^–^
Ac-AAPHAAA-NH_2_ (**PHA**)	**[FeL]**^**2+**^	3.15(6)	7.64	N_Im_
	**[FeH**_–**1**_**L]**^**+**^	–4.49(3)		N_Im_, OH^–^
	**[FeH**_–**3**_**L]**^–^	–21.98(3)	9.66	N_Im_, 3OH^–^
	**[FeH**_–**4**_**L]**^**2**–^	–31.64(4)		N_Im_, 4OH^–^
Ac-AAPHPAA-NH_2_ (**PHP**)	**[FeL]**^**2+**^	3.30(5)	7.79	N_Im_
	**[FeH**_–**1**_**L]**^**+**^	–4.49(3)		N_Im_, OH^–^
	**[FeH**_–**3**_**L]**^–^	–22.28(2)	10.05	N_Im_, 3OH^–^
	**[FeH**_–**4**_**L]**^**2**–^	–32.33(3)		N_Im_, 4OH^–^

a Values in parentheses are standard deviations of the last significant figure.

For all of the studied systems, Fe(II) complexes begin to form above pH 4, as in the case of Zn(II). The first detected species is [FeL]^2+^, where the histidine residue is unprotonated and bound to the metal ion. With the increasing pH, four water molecules, which complete the Fe(II) coordination sphere, undergo stepwise deprotonation, resulting in [FeH_–1_L]^+^, [FeH_–3_L]^−^, and [FeH_–4_L]^2–^ complexes. The almost perfect correspondence between the speciation models of the three Fe(II)/peptide systems, especially in the case of the two ligands **AHA** and **PHP**, leads to the conclusion that after the anchoring of the metal ion to the His residue, no additional interaction with peptide ligands (and then with backbone amides) takes place.

### Comparison of Metal Chelating Abilities

**Figure 4:**
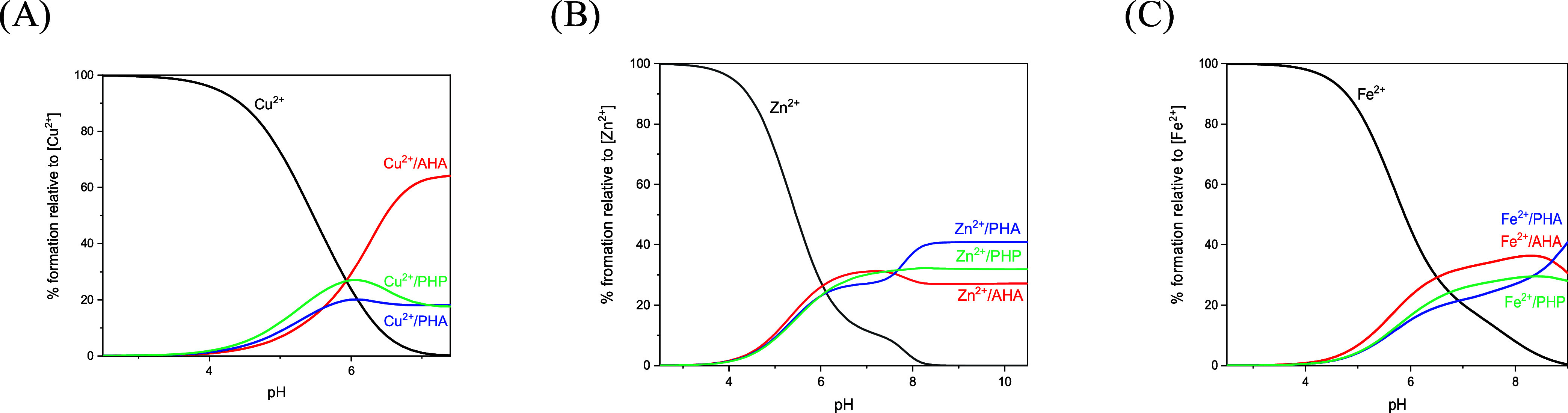
Competition plots for a simulated solution containing equimolar concentrations of (A) Cu(II), (B) Zn(II), (C) Fe(II), and **AHA**, **PHA**, and **PHP**. The diagrams are calculated from the overall stability constants of the binary complexes and simulate a situation in which equimolar concentrations of all of the chosen ligands and metals are present in a solution.

In an attempt to better understand the binding ability of the investigated ligands toward Cu(II), Zn(II), and Fe(II) ions, competition plots have been built up (Figures 4 and S13). These diagrams are calculated from the overall stability constants of the binary complexes and simulate a situation in which equimolar concentrations of all of the chosen ligands and metals are present in a solution; such competition diagrams are a powerful way to compare the complexing affinity of **AHA**, **PHA**, and **PHP** peptides toward the Cu(II), Zn(II), and Fe(II) ions for the whole explored pH range.

From the competition plot of copper complexes with the three peptides (Figure 4A), it is possible to notice how **AHA** leads to the formation of copper complexes with much higher stability than that of the other two peptides, especially at an alkaline pH. The substitution of one or two alanines adjacent to the metal-binding site with one or two prolines reduces the number of available amides of the peptide backbone for the complexation of the copper ion. In fact, the results reported above show that the copper ion can coordinate up to four nitrogens with the peptide **AHA**, while with **PHA** and **PHP**, the coordination stops at two or three nitrogens. The competition curve for the Cu(II)–PHP system has been stopped at pH 7.5 due to precipitation.

What is even more striking is the fact that the stabilities of the Zn(II) complexes are almost identical (Figure 4B). This proves that the coordination mode in the native AHA peptide does not differ from that of its Pro-substituted analogues’, clearly indicating that the coordination modes do not involve amide nitrogens.

The same situation is observed for Fe(II) complexes of our model systems: their stabilities are fairly similar; therefore, amide binding in the case of the AHA peptide can be excluded.

### Density Functional Theory (DFT) Studies Confirm (Lack of) Amide Coordinating Abilities

**Figure 5:**
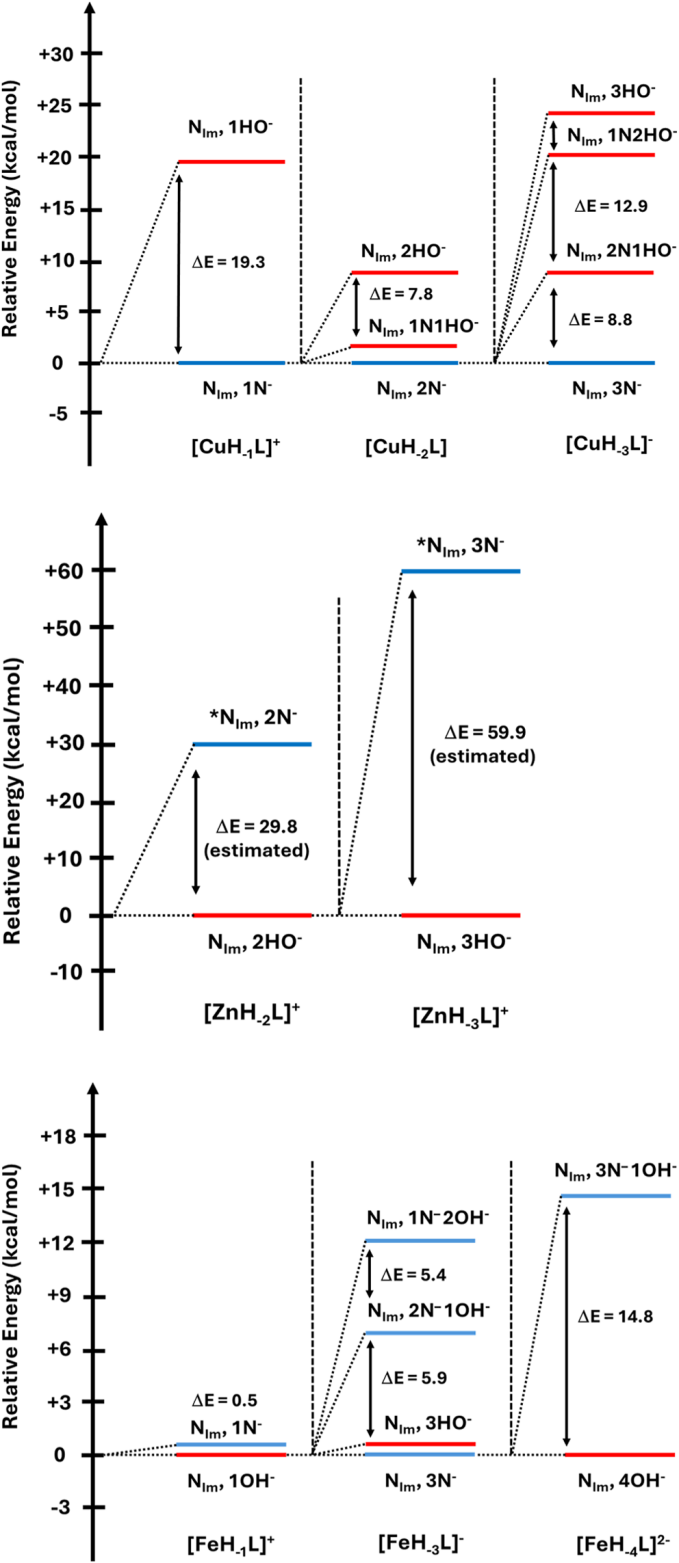
Correlative energies of mononuclear 1:1 metal–peptide MH_–*x*_L species formed as a function of the deprotonation degree with the increasing solution pH. In all cases, the relative energies of the complexes were evaluated for the OH^–^ versus the corresponding amide-deprotonated structures. The * indicates a nonconvergent structure whose energy was estimated by its lowest computed point on the potential energy surface.

Finally, in order to support the experimental results, we investigated computationally the relative energies of the mononuclear 1:1 metal–peptide MH_–*x*_L species formed as a function of the deprotonation degree with the increasing solution pH. In all cases, the relative energies of the complexes were evaluated for the OH^–^ vs the corresponding amide-deprotonated structures (Figure 5). For the peptide, stepwise amide deprotonation was evaluated as Ac-A^3^A^2^A^1^HAAA-NH_2_, where for A^#^, # indicates the sequence of deprotonated alanines.

In the case of the Cu(II) ion, the computational data are in agreement with the experimental results in that in all cases, with increasing pH value, amide deprotonation and coordination to copper(II) lead to more stable (N_Im_, nN^–^) complexes as compared to the corresponding hydroxy (N_Im_, *n*OH^–^) complexes ensuing from coordinated water deprotonation.

Considering that the Zn(II) ion binds to AHA in a 1:1 molar ratio, the computational data suggest that amide (N_Im_, *n*N^–^) complexes are unlikely to form in solution due to the very high strain imposed on the tetrahedral geometry of the ion and because of the fact that [ZnH_–*x*_L] complexes generated via the simple release of protons from the coordinated water molecules are the most likely species to be formed. Indeed, despite our efforts, amide-deprotonated [ZnH_–2_L] and [ZnH_–3_L]^−^ species never converged in the calculations.

Finally, the Fe(II) ion case proved to be the most challenging and more difficult to interpret. In its low-spin state, DFT calculations indicate that for [FeH_–1_L]^+^ and [FeH_–3_L]^−^ species, both amide (N_Im_, *n*N^–^) and hydroxy (N_Im_, *n*OH^–^) complexes are very close in energy (only ca. 0.5 kcal/mol Δ*E*), while at high pH, [FeH_–4_L]^2–^, in which four coordinated water molecules are deprotonated, is far more stable than the corresponding (N_Im_, 3N, 1OH^–^) complex. Overall, considering both theoretical and experimental evidence, we conclude that although theoretically possible, it is highly unlikely that backbone amide deprotonation is the metal-anchoring site for the Fe(II) ion under experimental conditions.

## Conclusions

In the present study, for the first time, precise details of the formation equilibria and metal coordination modes of Cu(II), Zn(II), and Fe(II) complexes with the model peptide Ac-AAAHAAA-NH_2_ and two mutants have been deeply investigated in order to obtain a clear answer of the capability of Zn(II) and Fe(II) to displace backbone amide protons and bind amide nitrogens, as Cu(II) notoriously does. Both experimental and computational results clearly show that the metal-induced amide deprotonation and binding are not likely for the model Ac-AAAHAAA-NH_2_ peptide in the case of both Zn(II) and Fe(II) ions. As a matter of fact, the complex-formation behavior of Ac-AAAHAAA-NH_2_ toward Zn(II) and Fe(II) is nearly identical to that of its Ala-to-Pro substituted analogues, Ac-AAPHAAA-NH_2_ and Ac-AAPHPAA-NH_2_, where the tertiary nitrogen atoms of backbone amides belonging to proline residues around the histidine, the metal-anchoring site, cannot bind metal ions. This simple model study clarifies the lately ongoing discussion about possible Zn(II)- and Fe(II)-amide binding and constitutes a valuable contribution to the very sparse literature data on iron–peptide complexes.

## Experimental Section

### Materials and Methods

The peptides (Ac-AAAHAAA-NH_2_, Ac-AAPHAAA-NH_2_, and Ac-AAPHPAA-NH_2_) were purchased from KareBio and used as received. Copper(II), zinc(II) chloride, and ammonium iron(II) sulfate were extra pure products (Sigma-Aldrich). The carbonate-free stock solution of 0.1 M NaOH (Sigma-Aldrich) was potentiometrically standardized with potassium hydrogen phthalate (Sigma-Aldrich). In the case of iron(II), the concentration of Fe(II) ions was determined using UV–vis spectroscopy of the Fe(II)–phenanthroline complex, as published before.^9^

### Mass Spectrometry

High-resolution mass spectra were obtained on a Bruker ESI-Q-TOF spectrometer (Bruker Daltonics, Germany). The mass spectrometer was operated in positive- and negative-ion modes. The instrumental parameters were as follows: scan range was *m*/*z* 50–3000, dry gas was nitrogen, temperature was 180 °C, and ion energy was 5 eV. The capillary voltage was optimized to the highest S/N ratio, and it was 3500 V. The samples ([*L*]_tot_ = 1 × 10^–4^ M and M:L molar ratio = 0.9:1) were prepared in a 1:1 methanol–water mixture, and the initial pH was set to 7.4. The sample was infused at a flow rate of 180 μL min^–1^. The instrument was calibrated externally with a Low Concentration Tuning Mix ESI-FOF instrument (Agilent Technologies (SD ≤ 1 ppm) and sodium formate clusters (SD ≤ 1 ppm)). Data were processed using the Bruker Compass DataAnalysis 4.2 program. The mass accuracy for the calibration was >5 ppm, enabling, together with the true isotopic pattern, an unambiguous confirmation of the elemental composition of the obtained complex.

### Potentiometric Measurements

The stability constants for ligand protonation and metal complex formation were calculated from potentiometric titration curves carried out over the pH range of 2.0–11.0 at *T* = 298 K. The total volume of the solution was 3 mL in the case of Cu(II) and Zn(II) complexes and 1.5 mL in the case of Fe(II) complexes. The pH-metric titrations were performed with a Metrohm 809 Titrando pH-meter titrator provided with a Mettler-Toledo glass body, microcombination pH electrode. The titration cell was equipped with a magnetic stirring system, a microburet delivery tube, and an inlet–outlet tube for high-purity grade argon in order to maintain an inert atmosphere. Solutions were titrated with 0.1 M carbonate-free NaOH. The electrode was calibrated daily for hydrogen ion concentration by titrating HCl with NaOH in the same experimental conditions as above. Purities and the exact concentrations of the ligand solutions were determined by the Gran method.^35^ The ligand concentration was 0.5 mM, and the metal-to-ligand ratio was 0.9:1. Due to the high susceptibility of Fe(II) ions to oxidation, all of the experiments were carried out in degassed solvents and under anaerobic conditions according to the procedure described in our previous paper.^9^ Argon provides an inert atmosphere and prevents the oxidation and diffusion of CO_2_ to the solution. It is worth noting that during the experiment, no color changes of the test sample or iron hydroxide precipitation were observed until pH 9.5. Access to the atmospheric oxygen resulted in the sample color turning yellow, followed by the precipitation of a brown-yellow Fe(III) hydroxide. The standard potential and the slope of the electrode couple were computed by means of the Glee program.^36^ The HYPERQUAD2013 program was used for calculating the stability constants.^37^ The constants for hydrolytic of Cu(II), Zn(II), and Fe(II) species were used in these calculations and taken from the literature.^38,39^ The speciation and competition diagrams were computed with the HySS program^40^ and drawn in the OriginPro 2016 program.

### Calorimetric Measurements

Protonation and metal complex-formation enthalpy (Δ*H°*) values were determined for the three peptides and their Cu(II) and Zn(II) complexes by titration calorimetry, with a Tronac model 450 isoperibol calorimeter, equipped with a 3 mL reaction vessel. The corresponding data for the Fe(II) complexes cannot be determined with this system, since it is not possible to avoid contact with the atmosphere during the experiments. The calorimetric measurements were carried out by titrating aliquots of samples of the same composition as in potentiometry with a standard solution of HCl. The reaction heats—corrected for nonchemical contributions^41^ and for the dilution heats determined through specific experiments—were calculated by considering the calories as equivalent to 4.187 J. The protonation and complex-formation enthalpies and entropies were computed from experimental calorimetric titration by means of the computer program HypΔ*H*.^42^

### Spectroscopic Measurements

The absorption spectra of Cu(II) containing solutions were recorded on a Varian Cary50 Probe spectrophotometer in the range 200–800 nm, using a quartz cuvette with an optical path of 1 cm. In order to obtain information on the structure of the most abundant species in the solution, the observed wavelength of the maximum absorption at a given pH was compared with the expected λ_max_ value obtained from the literature.^12,21,25,43,44^ Circular dichroism (CD) spectra were recorded on a Jasco J-1500 spectropolarimeter in the 240–800 nm range. Electron paramagnetic resonance (EPR) spectra were recorded in liquid nitrogen on a Bruker ELEXSYS E500 CW-EPR spectrometer at X-band frequency (9.6 GHz) and equipped with an ER 036TM NMR teslameter and an E41 FC frequency counter. Ethylene glycol (30%) was used as a cryoprotectant. The EPR parameters were analyzed by computer simulation of the experimental spectra using WIN-EPR SIMFONIA software, version 1.2 (Bruker, Billerica, MA).

### DFT Calculations

All computations were performed with Gaussian 09 programs. Geometry optimizations and frequency calculations were performed in the water. The B3LYP functional was used in combination with the standard 6-31G(d,p) basis sets. The spin state of the Cu(II) and Fe(II) ions were set, respectively, as doublet and singlet. The default spin formalism was followed in the calculations, and default Gaussian 09 values were adopted for the numerical integration grids, self-consistent-field (SCF), and geometry optimization convergence criteria. Geometries were optimized without symmetry restrictions. The nature of the stationary points was checked by computing the vibrational frequencies to verify true minima. The analysis also provided all thermochemical quantities used to calculate the relative energy of the different M(II)-AHA species at different deprotonation states. No imaginary frequencies were observed in any of the computed species, except for the amide-deprotonated [ZnH_–2_L] and [ZnH_–3_L]^−^ species, which never converged.

To calculate the relative energies of the OH^–^ vs the corresponding amide-deprotonated M(II)–AHA structures, the stoichiometry was kept constant where possible.^45^ Alternatively, the relative energy of the deprotonated M(II)–AHA complexes was calculated considering the thermochemical data of the Gaussian output by adopting the free energies of reaction changes (Δ_*r*_*G*°) according to^46^
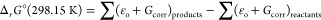
where ε_o_ = total energy of the molecule and *G*_corr_ = Gibbs free energy correction, for the following general reaction, considering the entropy increase of the liberated water molecules:
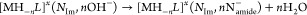


In the above reaction, *n*OH^–^ and *n*N_amide_^–^ are the number of deprotonated water molecules and the number of deprotonated amides, respectively, directly coordinated to the metal ion M. All energies are given in kcal/mol calculated according to E = Hartrees * 627.5095.

## Supporting Information Available

ESI-MS spectra, species distribution diagrams, thermodynamic parameters of protonation, and metal complex formation for all studied complexes; EPR parameters for Cu(II) complexes; and competition plots (PDF)
