# Supplementary material for: Metal-Induced Amide Deprotonation and Binding Typical for Cu(II), Not Possible for Zn(II) and Fe(II)
Source: Inorg Chem. 2025 Mar 26;64(13):6751–60. doi: 10.1021/acs.inorgchem.5c00672 (PMC11979888; doi:10.1021/acs.inorgchem.5c00672)
Supplement: Supplementary file 1 — ic5c00672_si_001.pdf [file ic5c00672_si_001.pdf]

Supplementary Information

Metal-induced amide deprotonation and binding – typical for Cu(II), not possible for Zn(II) and Fe(II)

Silvia Leveraro[a,b], Valentyn Dzyhovskyi[a], Kinga Garstka[a], Agnieszka Szebesczyk[c], Fabio Zobi[d], Denise Bellotti[d], Kamila Stokowa-Sołtys[a]*, Maurizio Remelli[d]*, Magdalena Rowińska-Żyrek[a]**

a) Faculty of Chemistry, University of Wroclaw, ul. F. Joliot-Curie 14, 50-383 Wroclaw, Poland; kamila.stokowa-soltys@uwr.edu.pl, magdalena.rowinska-zyrek@uwr.edu.pl

b) Department of Chemical, Pharmaceutical and Agricultural Sciences, University of Ferrara, Via Luigi Borsari 46, 44121 Ferrara, Italy; blldns@unife.it, rmm@unife.it

c) Institute of Health Sciences, University of Opole, Katowicka str. 68, 45-060 Opole, Poland

d) Department of Chemistry, Fribourg University, Chemin Du Musée 9, 1700, Fribourg, Switzerland

1. Mass spectrometry

A)

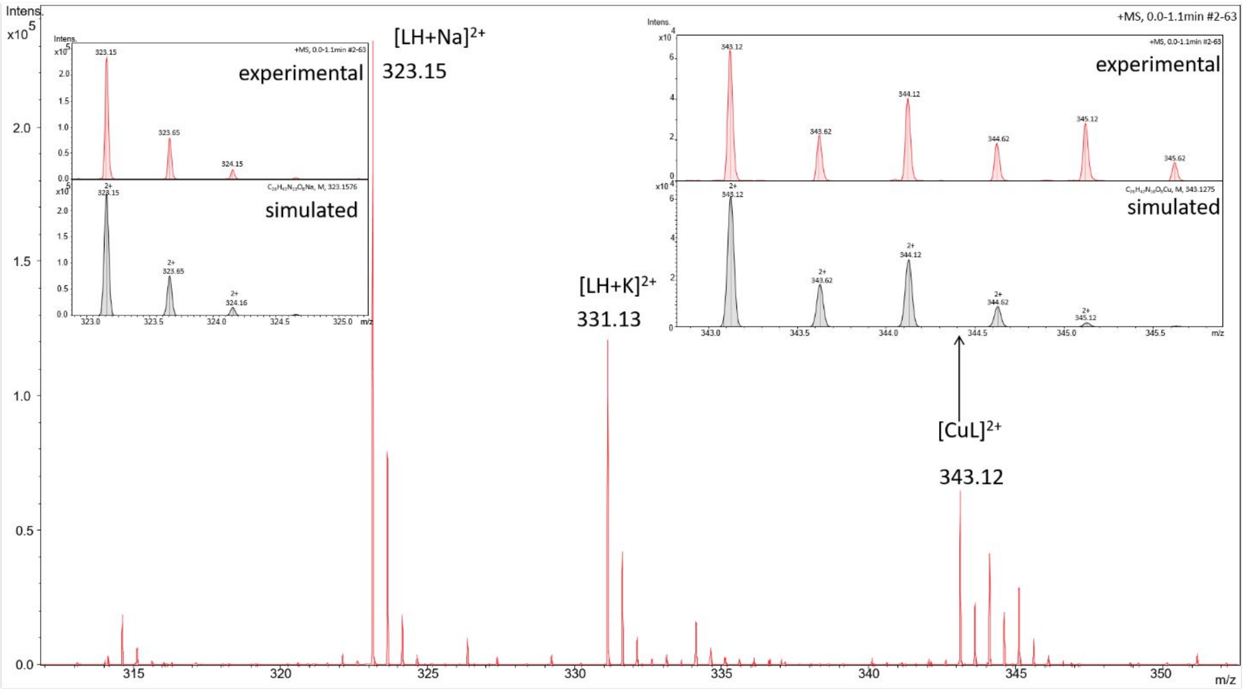

B)

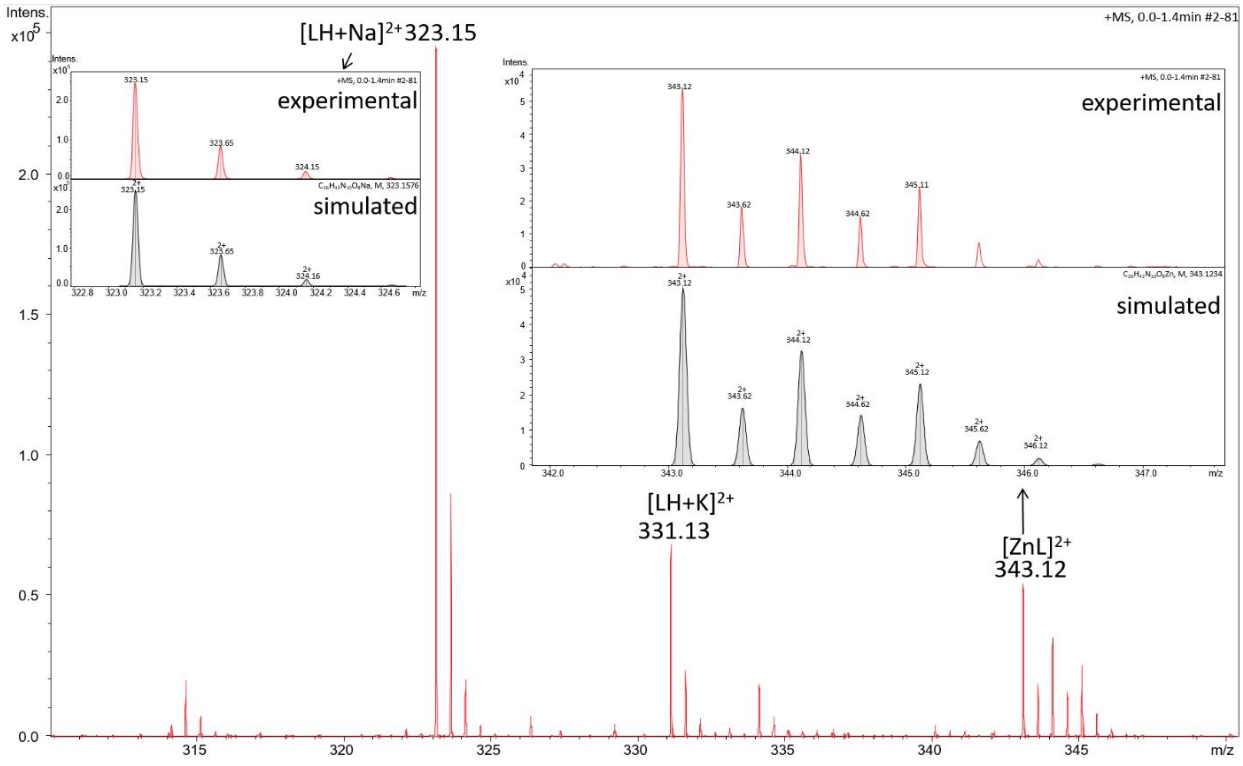

C)

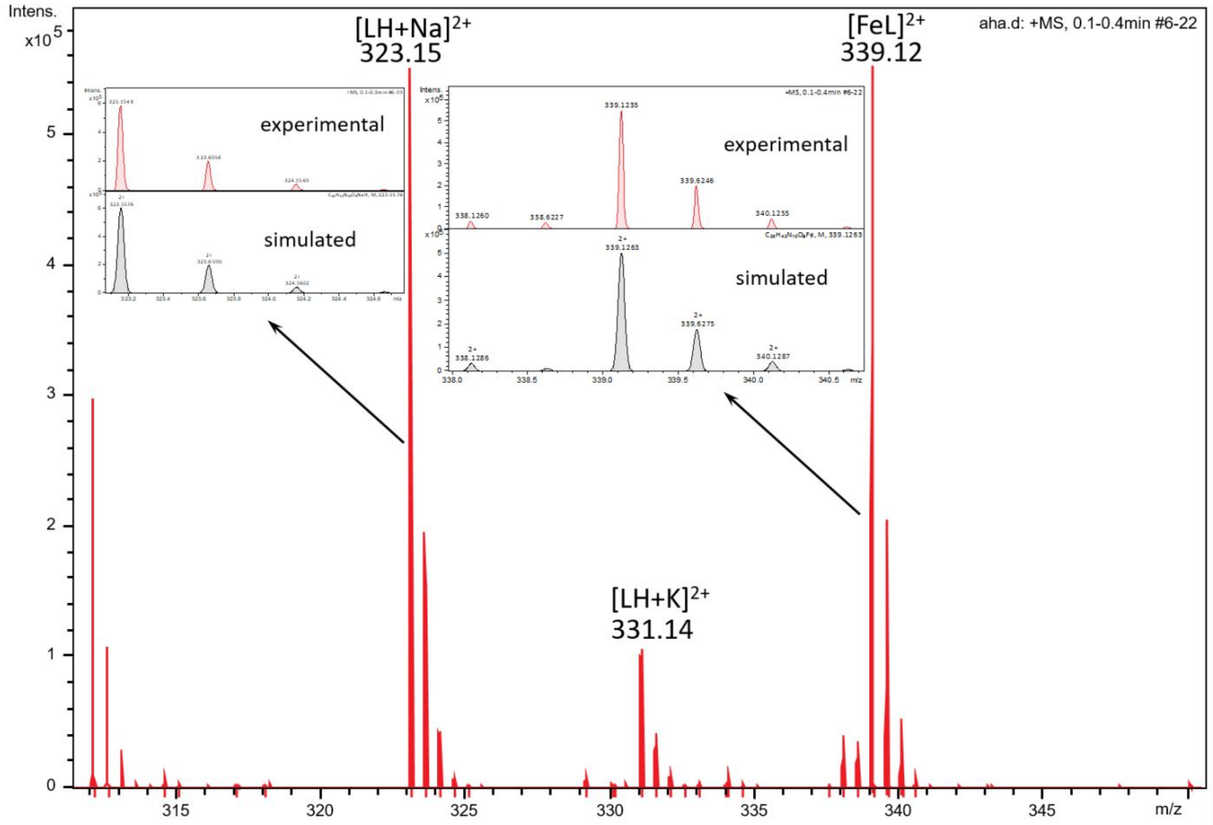

Figure S1. ESI-MS spectra for **AHA** containing systems, at L:M molar ratio=1:0.9 in MeOH:H_2O (1:1) mixture solution, and comparison between the experimental and simulated isotopic patterns of selected species. (A) Cu(II)/**AHA**; (B) Zn(II)/**AHA**; (C) Fe(II)/**AHA** system.

A)

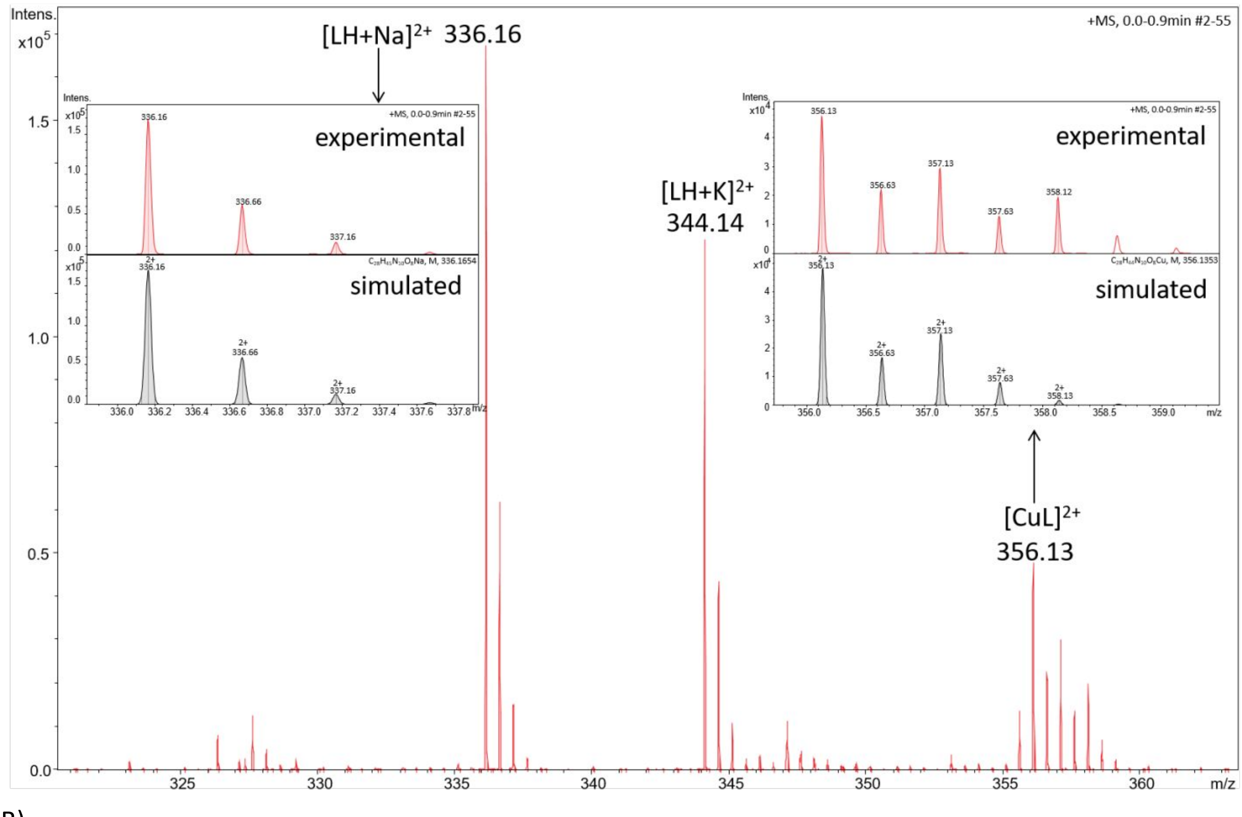
Intens.
x10^5
+MS, 0.0-0.9min #2-55
[LH+Na]2+ 336.16
experimental
simulated
[LH+K]2+
344.14
[CuL]2+
356.13
m/z

B)

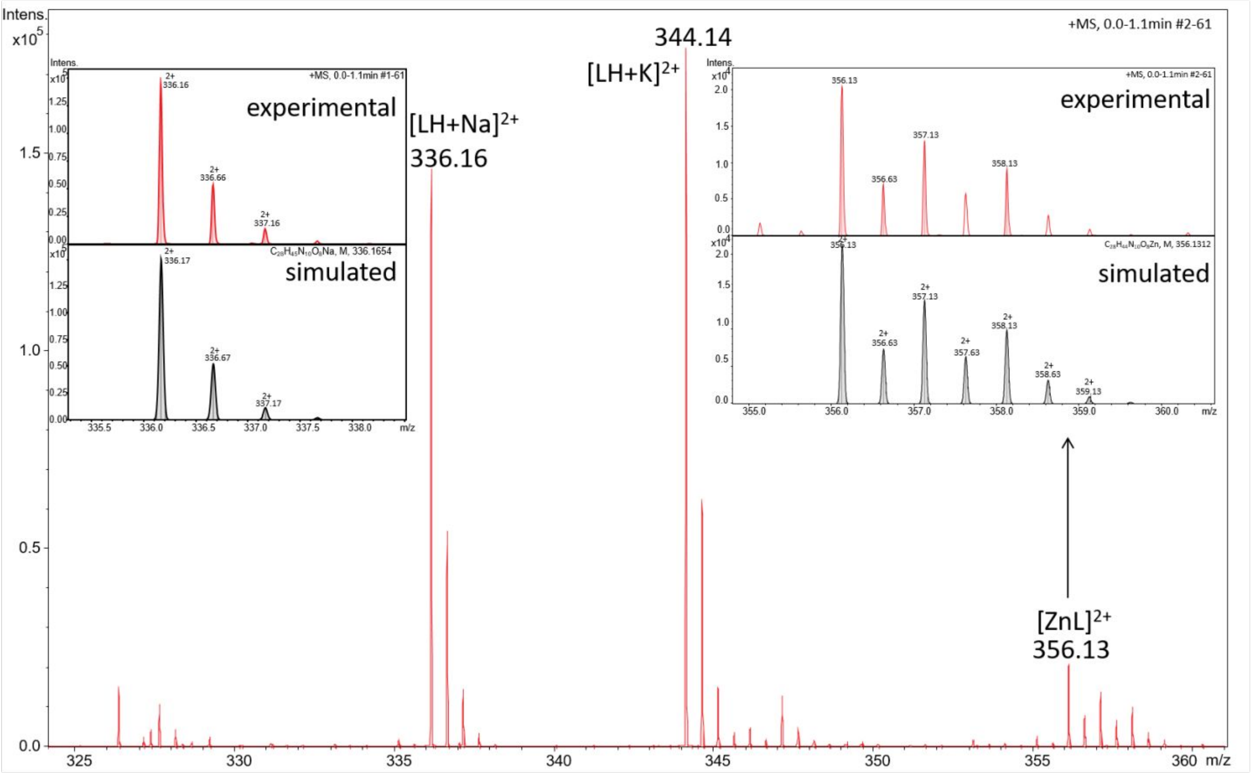

C)

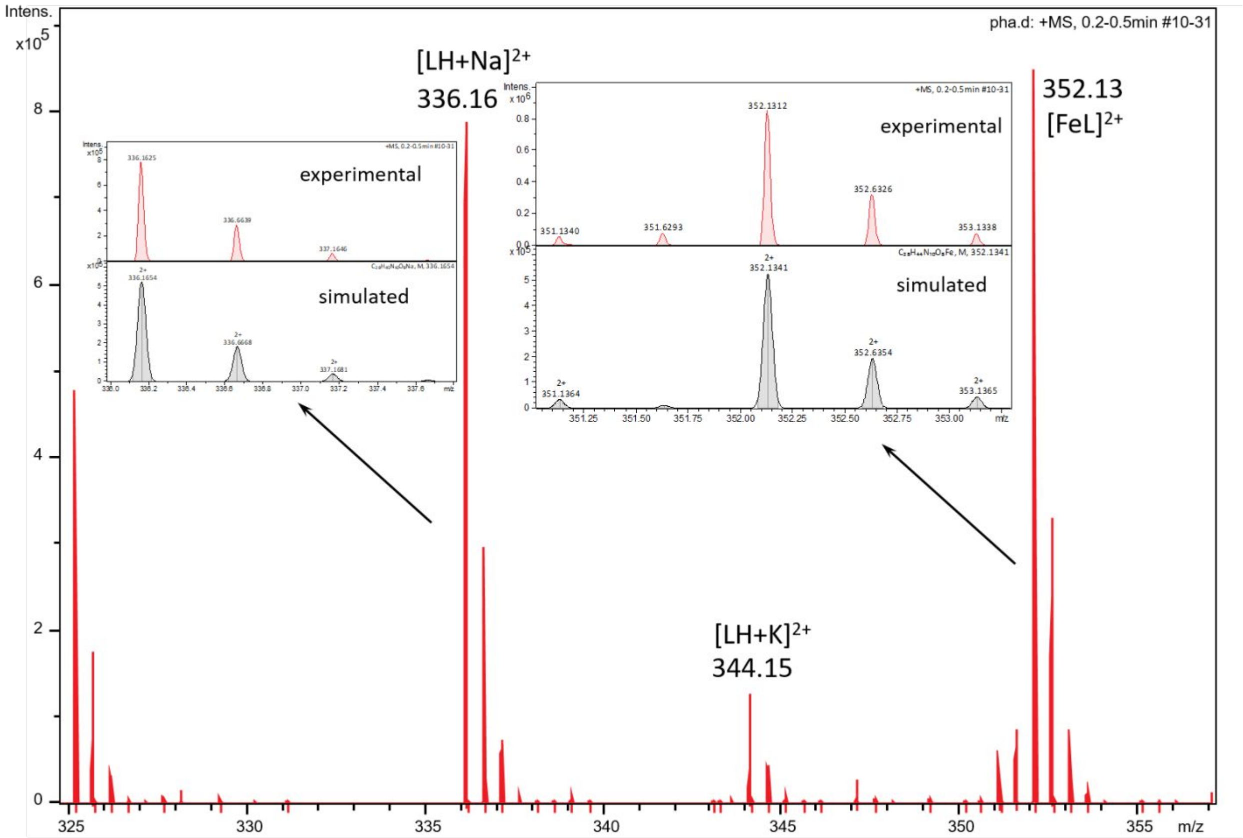

Figure S2. ESI-MS spectra for **PHA** containing systems, at L:M molar ratio=1:0.9 in MeOH:H_2O (1:1) mixture solution, and comparison between the experimental and simulated isotopic patterns of selected species. (A) Cu(II)/**PHA**; (B) Zn(II)/**PHA**; (C)Fe(II)/**PHA** system.

A)

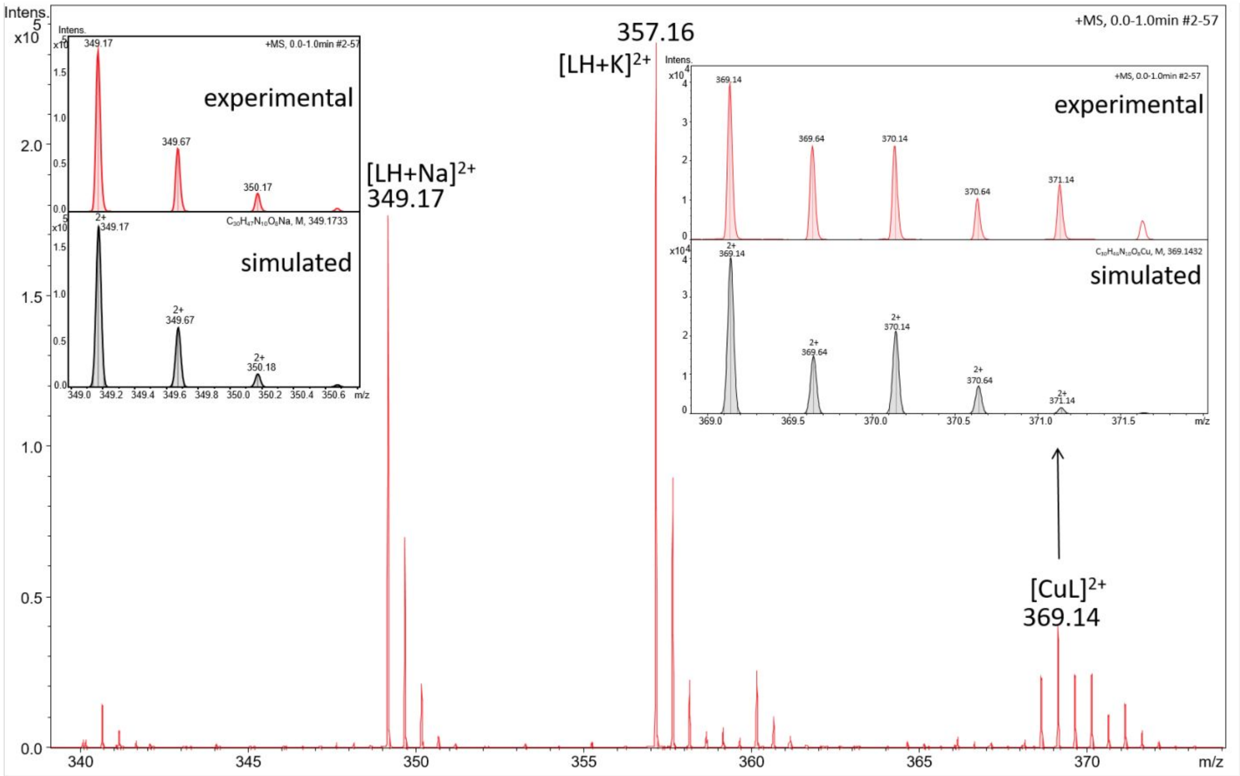

Intens.
x10^5
+MS, 0.0-1.0min #2-57
357.16
[LH+K]2+
[LH+Na]2+
349.17
experimental
simulated
[CuL]2+
369.14
349.17
349.67
350.17
350.18
369.64
370.14
370.64
371.14
m/z

B)

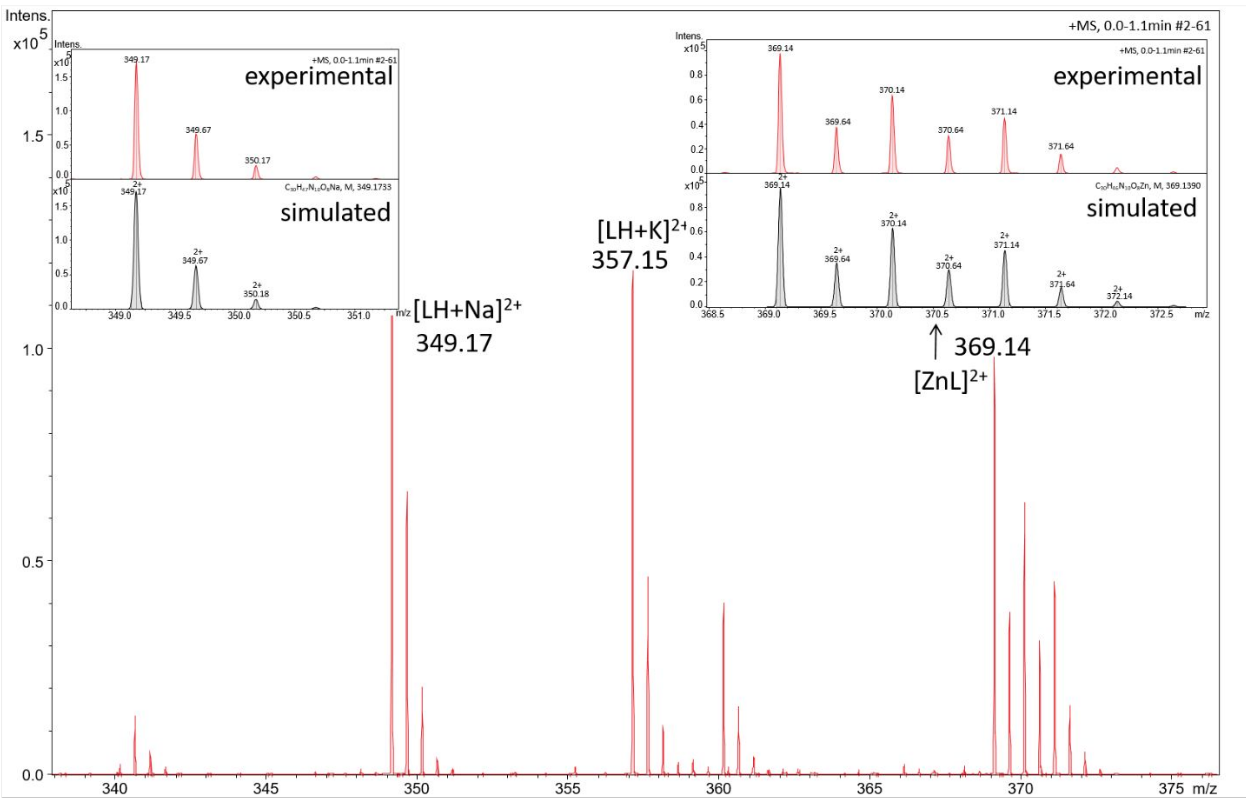

Intens.
x10^5
+MS, 0.0-1.1min #2-61
experimental
simulated
[LH+K]2+
357.15
[LH+Na]2+
349.17
369.14
[ZnL]2+
349.67
350.17
350.18
369.64
370.14
370.64
371.14
371.64
372.14
m/z

C)

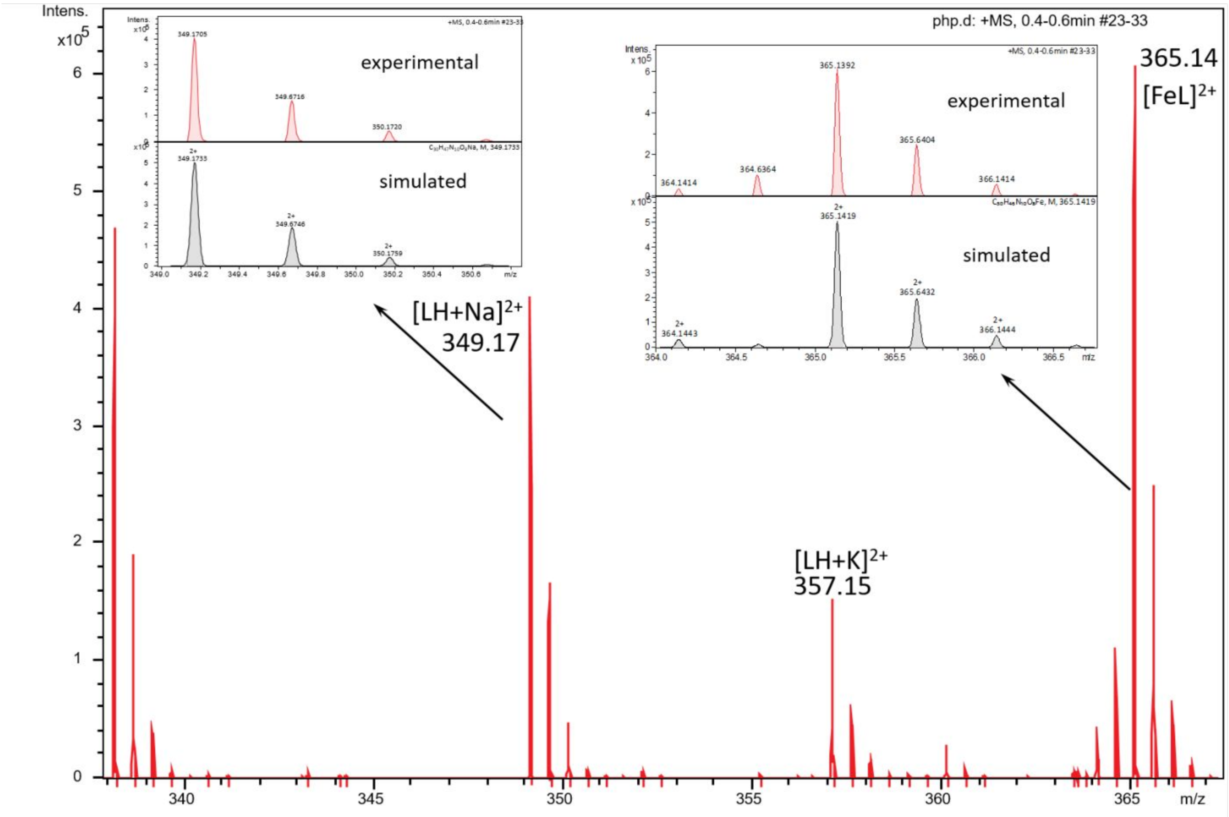

Figure S3. ESI-MS spectra for **PHP** containing systems, at L:M molar ratio=1:0.9 in MeOH:H_2O (1:1) mixture solution, and comparison between the experimental and simulated isotopic patterns of selected species. (A) Cu(II)/**PHP**; (B) Zn(II)/**PHP**; (C)Fe(II)/**PHP** system.

2. Speciation diagrams of metal complexes

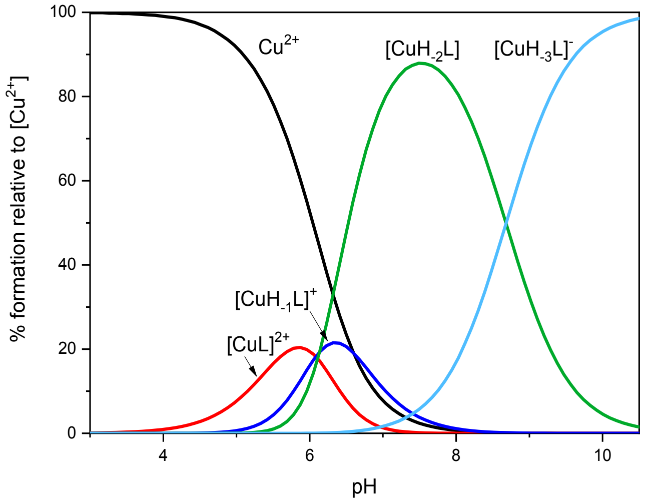

Figure S4. Exemplificative species distribution diagram for the system Cu(II) / **AHA**. Ligand concentration: 0.5 mM; M:L ratio 0.9:1.

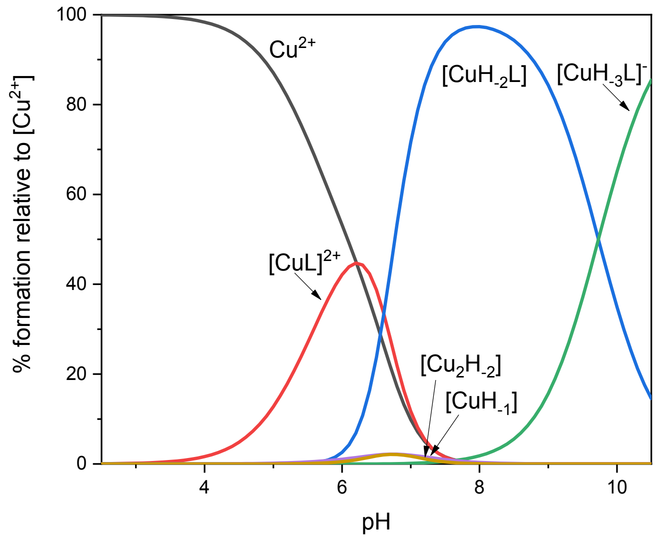

Figure S5. Exemplificative species distribution diagram for the system Cu(II) / **PHA**. Ligand concentration: 0.5 mM; M:L ratio 0.9:1.

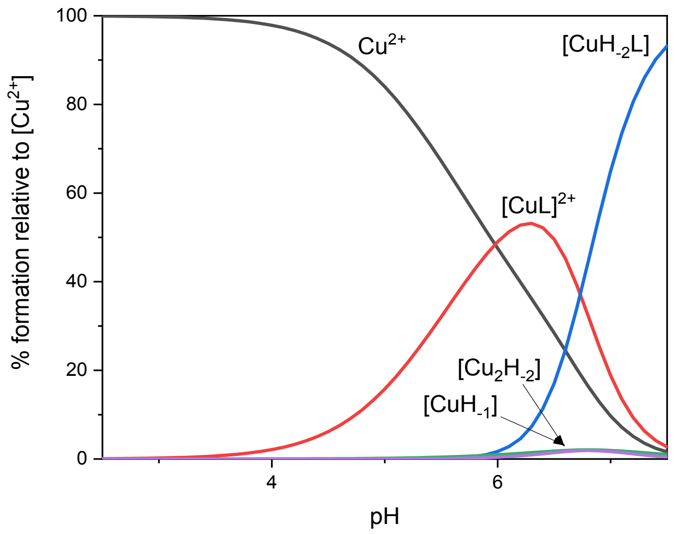

Figure S6. Exemplificative species distribution diagram for the system Cu(II) / **PHP**. Ligand concentration: 0.5 mM; M:L ratio 0.9:1.

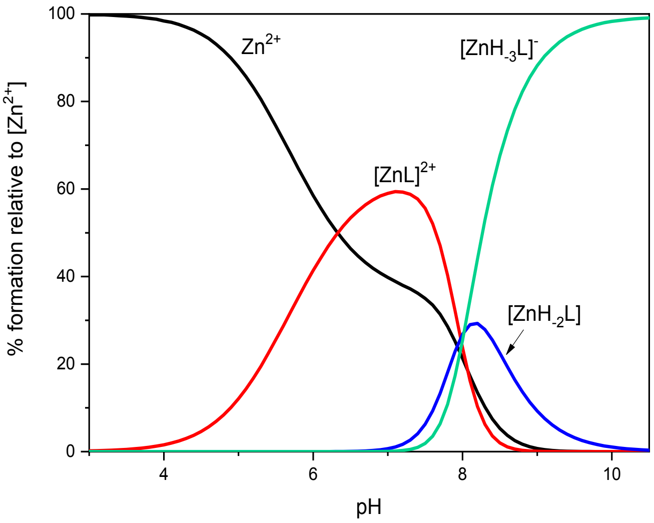

Figure S7. Exemplificative species distribution diagram for the system Zn(II) / **AHA**. Ligand concentration: 0.5 mM; M:L ratio 0.9:1.

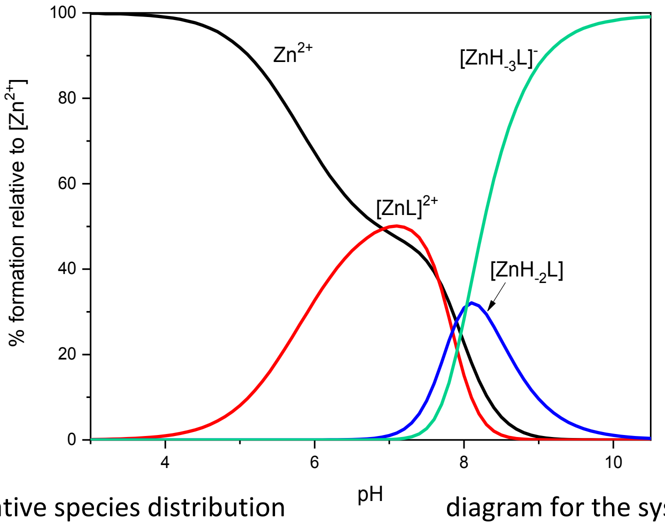

Figure S8. Exemplificative species distribution diagram for the system Zn(II) / **PHA**. Ligand concentration: 0.5 mM; M:L ratio 0.9:1.

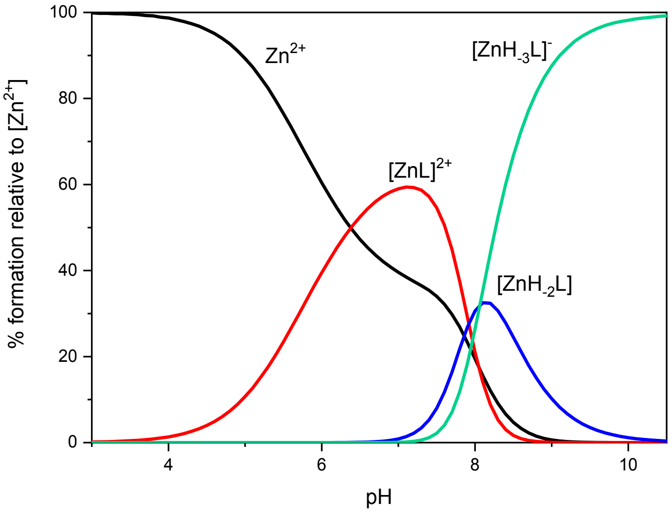

Figure S9. Exemplificative species distribution diagram for the system Zn(II) / **PHP**. Ligand concentration: 0.5 mM; M:L ratio 0.9:1.

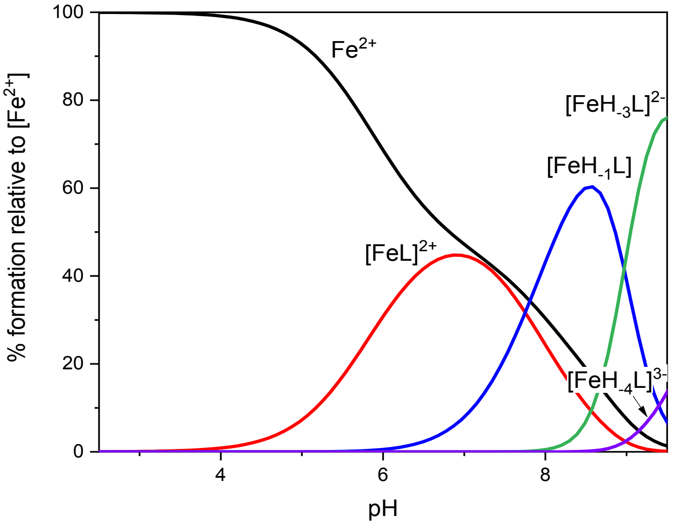

Figure S10. Exemplificative species distribution diagram for the system Fe(II) / **AHA**. Ligand concentration: 1mM; M:L ratio 0.9:1.

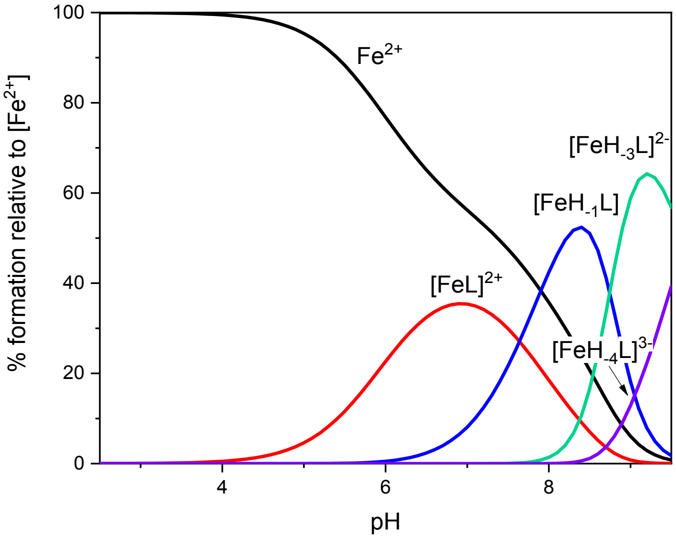

Figure S11. Exemplificative species distribution diagram for the system Fe(II) / **PHA**. Ligand concentration: 1mM; M:L ratio 0.9:1.

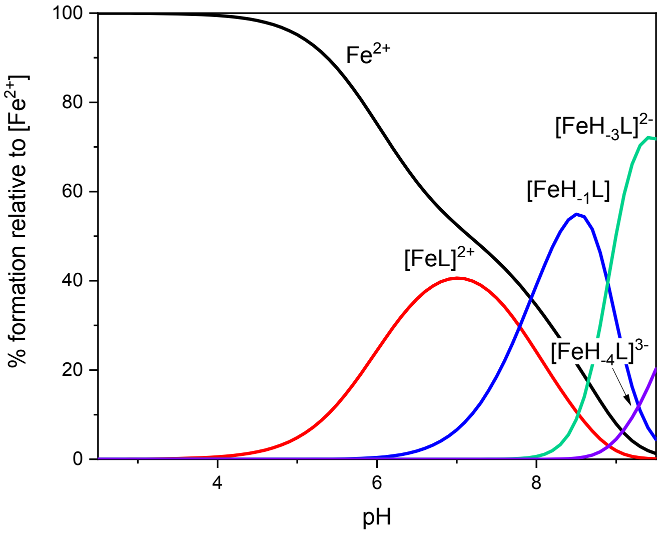

Figure S12. Exemplificative species distribution diagram for the system Fe(II) / **PHP**. Ligand concentration: 1mM; M:L ratio 0.9:1.

3. Calorimetric data

Table S1. Thermodynamic parameters of protonation and metal complex formation for the three peptides under investigation, at 25 °C and *I* = 0.1 M (KCl). Errors are reported as standard deviation on the last significant digit.

AHA

Species	log *β*	-Δ*G*° (kJ/mole)	-Δ*H*° (kJ/mole)	Δ*S*° (J/(mole K))
HL^+	6.47(1)	36.9(1)	26.1(2)	36(1)
$[CuL]^{2+}$	3.44(6)	19.6(3)	22.4(3)	-9(2)
$[CuH_{-1}L]^+$	-2.63(3)	-15.0(2)	-9.5(3)	-18(1)
$[CuH_{-2}L]$	-8.77(1)	-50.0(1)	-43(1)	-24(4)
$[CuH_{-3}L]^-$	-17.45(2)	-99.5(1)	-73(2)	-89(8)
$[ZnL]^{2+}$	3.70(6)	21.1(3)	-13(4)	117(14)
$[ZnH_{-2}L]$	-12.27(6)	-70.0(3)	-66(5)	-12(18)
$[ZnH_{-3}L]^-$	-20.29(4)	-115.7(2)	-107(6)	-28(21)

PHA

Species	log *β*	-Δ*G*° (kJ/mole)	-Δ*H*° (kJ/mole)	Δ*S*° (J/(mole K))
HL^+	6.43(1)	36.7(1)	23.2(2)	45(1)
$[CuL]^{2+}$	3.56(4)	20.3(2)	17.4(3)	10(1)
$[CuH_{-2}L]$	-9.65(2)	-55.0(1)	-49(1)	-22(4)
$[CuH_{-3}L]^-$	-19.38(2)	-110.5(1)	-76(2)	-115(8)
$[ZnL]^{2+}$	3.6(1)	20.5(6)	-9(4)	99(15)
$[ZnH_{-2}L]$	-12.0(1)	-68.5(6)	-62(5)	-21(18)
$[ZnH_{-3}L]^-$	-20.01(8)	-114.1(5)	-103(6)	-38(21)

PHP

Species	log *β*	-Δ*G*° (kJ/mole)	-Δ*H*° (kJ/mole)	Δ*S*° (J/(mole K))
HL^+	6.56(2)	37.4(1)	23.6(2)	46(1)
$[CuL]^{2+}$	3.81(5)	21.7(3)	14.1(3)	26(1)
$[CuH_{-2}L]$	-9.66(3)	-55.1(2)	-37(1)	-61(4)
$[ZnL]^{2+}$	3.70(6)	21.1(3)	-7(4)	93(14)
$[ZnH_{-2}L]$	-12.13(6)	-69.2(3)	-55(5)	-47(18)

**$[ZnH_{-3}L]^{-}$**	-20.19(4)	-115.2(2)	-106(6)	-30(21)

4. EPR data

Table S2. EPR parameters for Cu(II) complexes with the studied peptides at *I*= 0.1 M (KCl) and M:L molar ratio = 0.9:1. C_L = 2.2 □10^{-3} M

pH	AHA					PHA					PHP				
	$A_{//}$ [G] (A_z)	$g_{//}$ (g_z)	$g_{\perp}$ (g_x=g_y)	MW Frequency [GHz]	Coordinated N	$A_{//}$ [G] (A_z)	$g_{//}$ (g_z)	$g_{\perp}$ (g_x=g_y)	MW Frequency [GHz]	Coordinated N	$A_{//}$ [G] (A_z)	$g_{//}$ (g_z)	$g_{\perp}$ (g_x=g_y)	MW Frequency [GHz]	Coordinated N
**3.0**	122	2.41	2.08	9.60	0	119	2.42	2.08	9.60	0	-	-	-	-	-
**4.0**	122	2.43	2.08	9.60	0	120	2.42	2.08	9.60	0	121	2.42	2.08	9.60	0
**5.0**	122	2.43	2.08	9.60	0	120	2.42	2.08	9.60	0	121	2.42	2.05	9.60	0
**6.0**	150	2.33	2.07	9.60	1	120	2.41	2.08	9.60	0	145	2.34	2.07	9.60	1
**7.0**	160	2.24	2.07	9.60	2/3	127	2.36	2.07	9.60	1	156	2.33	2.06	9.60	1
**8.0**	163	2.24	2.06	9.60	2/3	154	2.24	2.06	9.60	2	170	2.27	2.06	9.60	2
**9.0**	163	2.24	2.06	9.60	2/3	154	2.24	2.06	9.60	2	170	2.27	2.06	9.60	2
**10.0**	197	2.19	2.05	9.60	4	176	2.22	2.05	9.60	3	174	2.26	2.06	9.60	2
**11.0**	197	2.19	2.05	9.60	4	203	2.20	2.05	9.60	3/4	189	2.24	2.05	9.60	2

5. Comparison of metal chelating abilities

A)

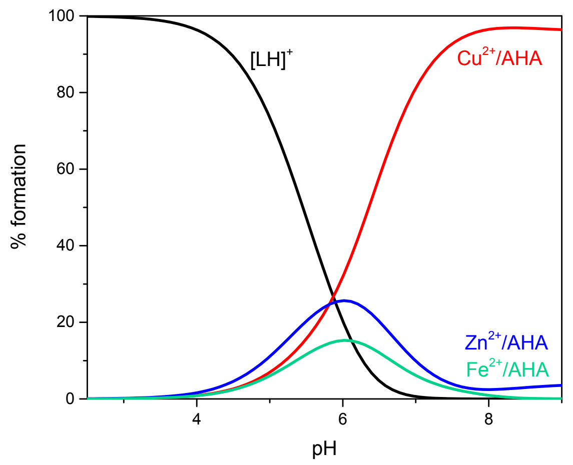

B)

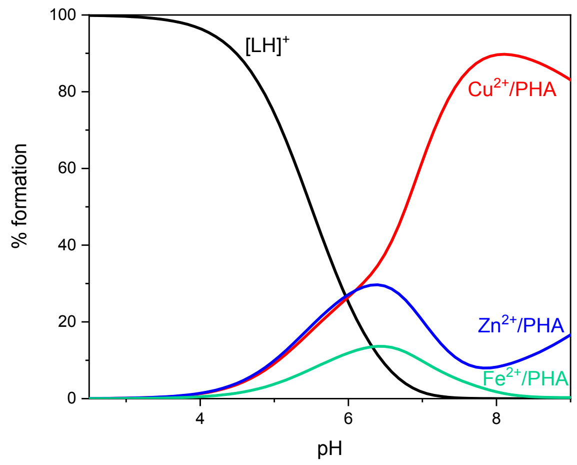

C)

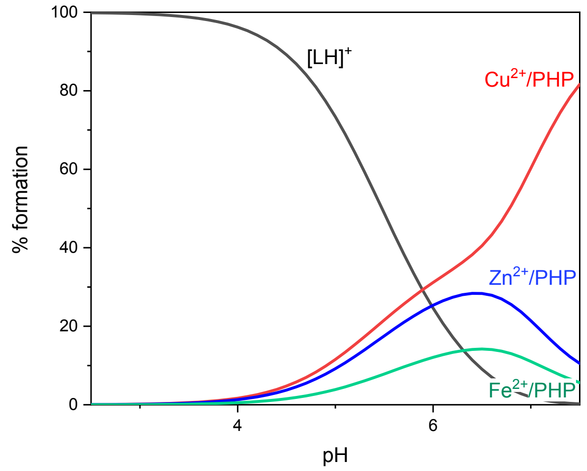

Figure S13. Competition plots for a simulated solution containing equimolar concentrations of (A) Cu(II), Zn(II), Fe(II), and **AHA** , (B) Cu(II), Zn(II), Fe(II), and **PHA**, (C) Cu(II), Zn(II), Fe(II), and **PHP.**
